# Crystal structure of bis­(ethyl­enedi­thio)­tetra­thia­fulvalenium μ_2_-acetato-bis­[tri­bromido­rhenate(III)] 1,1,2-tri­chloro­ethane hemisolvate

**DOI:** 10.1107/S2056989016006058

**Published:** 2016-04-19

**Authors:** Alexander A. Golichenko, Andrey V. Kravchenko, Irina V. Omelchenko, Denis M. Chudak, Vladimir A. Starodub, Boleslaw Barszcz, Alexander V. Shtemenko

**Affiliations:** a Department of Inorganic Chemistry, Ukrainian State University of Chemical Technology, Gagarin Ave. 8, Dnipropetrovsk 49005, Ukraine; b Applied Chemistry Department, V. N. Karazin Kharkiv National University, 4 Svoboda Square, Kharkiv, 61022, Ukraine; c SSI "Institute for Single Crystals" NAS of Ukraine, 60 Nauky Ave., Kharkiv, 61072, Ukraine; d Institute of Chemistry, Jan Kochanowski University, 25-406 Kielce, Poland; e Institute of Molecular Physics, Polish Academy of Sciences, 60-179 Poznan, Poland

**Keywords:** crystal structure, radical cation salt, bis­(ethyl­enedi­thio)­tetra­thia­fulvalene, rhenium, quadruple metal–metal bond

## Abstract

The asymmetric unit of the title salt, (C_10_H_8_S_8_)[Re_2_Br_6_(CH_3_COO)]·0.5C_2_H_3_Cl_3_, contains one bis­(ethyl­enedi­thio)­tetra­thia­fulvalene (ET) radical cation, one μ_2_-acetato-bis­[tri­bromido­rhenate(III)] anion and a 1,1,2-tri­chloro­ethane mol­ecule with half-occupancy disordered about a twofold rotation axis. The tetra­thia­fulvalene fragment adopts an almost planar configuration typical of the ET radical cation. The C atoms of both ethyl­enedi­thio fragments in the cation are disordered over two orientations with occupancy factors 0.65:0.35 and 0.77:0.23. In the anion, six Br atoms and a μ_2_-acetate ligand form a strongly distorted cubic O_2_Br_6_ coordination polyhedron around the Re_2_ dinuclear centre. In the crystal, centrosymmetrically related ET cations and Re_2_O_2_Br_6_ anions are linked into dimers by π–π stacking inter­actions [centroid-to-centroid distance = 3.826 (8) Å] and by pairs of additional Re⋯Br contacts [3.131 (3) Å], respectively. The dimers are further packed into a three-dimensional network by non-directional inter­ionic electrostatic forces and by C—H⋯Br and C—H⋯S hydrogen bonds. The disordered 1,1,2-tri­chloro­ethane mol­ecules occupy solvent-accessible channels along the *b* axis.

## Abstract 2

The crystal structure of a binuclear mono­carboxyl­ato dirhenium(III) complex with a fulvalene derivative is reported. This compound represents a radical cation salt containing a cluster unit with rhenium–rhenium quadruple bond.

## Chemical context   

In the past few decades, mol­ecular low-dimensional conducting materials have attracted much inter­est owing to their physical properties, in particular their electrical, magnetic and spectroscopic properties. The packing of radical cations in the crystal and the properties of radical cation salts depend substanti­ally on the type of anions involved (Mori *et al.*, 1999 ▸; Mori, 1999 ▸). Labile equatorial chloride or bromide groups around the Re_2_^6+^ cluster unit are the reactive centres in inter­actions with other chemical compounds and biological macromolecules (Shtemenko *et al.*, 2013 ▸, 2015 ▸). Only one radical cation salt containing a rhenium–rhenium quadruple bond has been described so far {(ET)_2_[Re_2_Cl_8_] [ET = bis(ethyl­enedi­thio)­tetra­thia­fulvalene]; Reinheimer *et al.*, 2008 ▸}. In this context, we present the synthesis and crystal structure of a new radical cation salt of ET with the dirhenium(III) anion [Re_2_Br_6_(CH_3_COO)]^−^. Neither acetic acid nor acetate was used in the synthesis of this radical cation salt. Evidently, the acetate ligand arose by hydrolysis of CH_3_CN (Cotton *et al.*, 1991 ▸). Complex compounds of dirhenium(III) with one equatorial carboxyl­ato ligand are not well studied, the structure of only three such rhenium compounds having been reported to date (Lau *et al.*, 2000 ▸; Vega *et al.*, 2002 ▸; Beck & Zink, 2011 ▸).
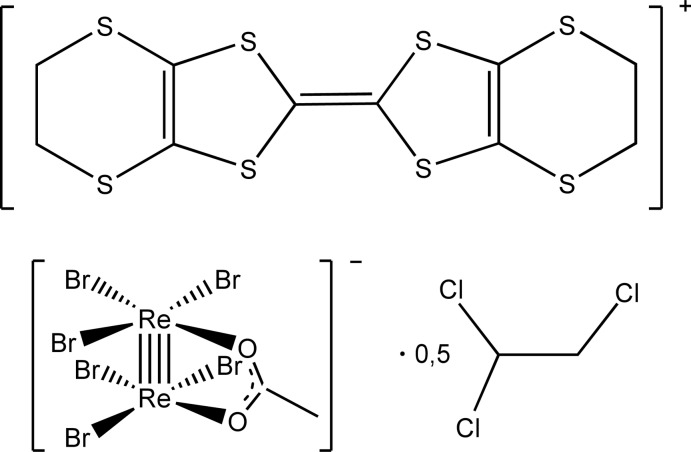


## Structural commentary   

**Figure 1:**
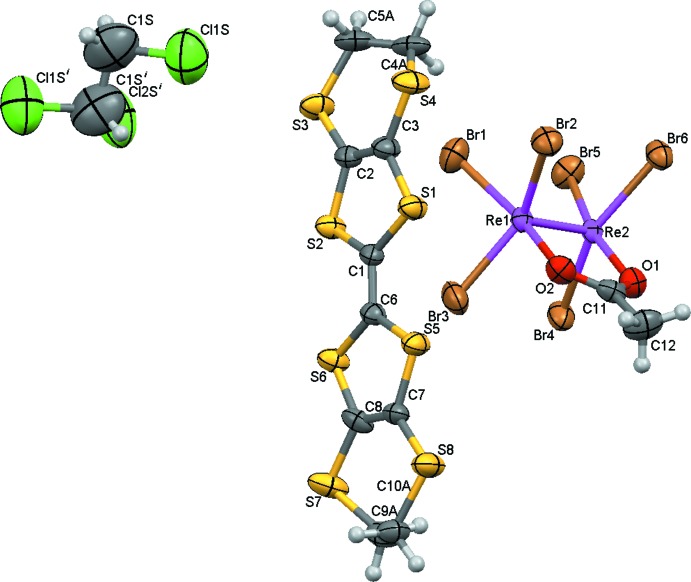
The asymmetric unit of the title compound, with displacement ellipsoids drawn at the 50% probability level. [Symmetry code: (i) −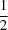 − *x*, *y*, −*z*.] Only one component of the disordered 1,1,2-tri­chloro­ethane mol­ecule and the major component of the ET cation are shown. Colour codes: C, grey; H, white; O, red; S, yellow; Cl, green; Br, brown, Re, violet.

The title compound (Fig. 1 ▸) consists of bis­(ethyl­enedi­thio)­tetra­thia­fulvalene (ET) radical cations, μ_2_-acetato-bis­[tri­bromido­rhenate(III)] anions and 1,1,2-tri­chloro­ethane mol­ecules in the stoichiometric molar ratio of 1:1:0.5. The solvent mol­ecule is disordered over two orientations of equal occupancy about a twofold rotation axis inter­secting the mid-point of the C—C ethane bond. The tetra­thia­fulvalene fragment adopts an almost planar configuration (r.m.s. deviation = 0.033 Å) that is typical for ET radical cations. The dihedral angle between the five-membered rings is 0.3 (6)°. The carbon atoms of both ethyl­enedi­thio fragments (C4/C5 and C9/C10) are disordered over two sets of sites with occupancy ratios of 0.65:0.35 and 0.77:0.23, respectively.

In the anion, each Re^III^ atom is coordinated by three Br atoms forming ReBr_3_ units which are linked by a Re—Re multiple bond [2.2174 (10) Å] and a bridging μ_2_-acetate ligand, forming a strongly distorted cubic O_2_Br_6_ coordination polyhedron around the Re_2_ core. The length of the Re—Re bond is very close to the mean value of 2.222 Å for quadruple bonds (Groom *et al.*, 2016 ▸), and the six bromine ligands are arranged into an eclipsed conformation. It is also known that the presence of *O*,*O*-bridging ligands in such structures has a negligible effect on the Re—Re bond length [it varies in the range 2.2067 (7)–2.2731 (9) Å for compounds with no bridging ligands and in the range 2.2168 (8)–2.2532 (2) Å for compounds with *O*,*O*-bridging ligands (Poineau *et al.*, 2015 ▸)]. Thus, the structure of the Re_2_Br_6_CH_3_COO^−^ anion corres­ponds to the typical structure of compounds with quadruple Re—Re bonds in an Re_2_^6+^ core (Cotton *et al.*, 2005 ▸). The Re—Br and Re—O bonds vary in the ranges 2.435 (3)–2.451 (3) Å and 2.009 (15)–2.040 (16) Å, respectively. The distortion from an ideal cubic geometry is mainly due to the short distance between the O atoms of the acetate group [2.24 (2) Å], while the Br⋯Br separations between adjacent Br atoms vary in the range 3.411 (3)–3.553 (4) Å.

## Supra­molecular features   

**Figure 2:**
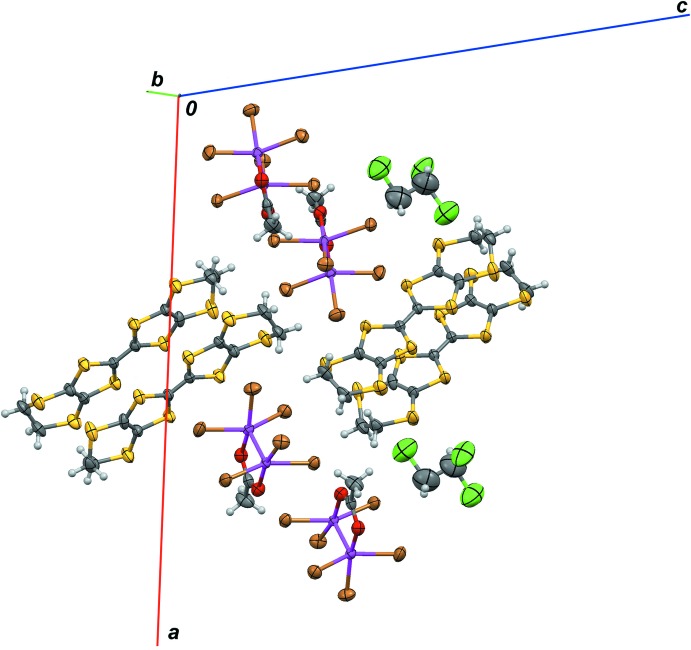
Partial crystal packing of the title compound, with displacement ellipsoids shown at the 50% probability level. Only one component of the disordered 1,1,2-tri­chloro­ethane mol­ecule and the major component of the ET mol­ecule are shown. Colour codes: C, grey, H, white, O, red, S, yellow, Cl, green, Br, brown, Re, violet.

In the crystal (Fig. 2 ▸), pairs of centrosymmetrically related ET cations are linked in a ‘head-to-tail’ manner into dimers by π–π stacking inter­actions, with centroid-to-centroid separations of 3.836 (8) Å, perpendicular inter­planar distances of 3.518 (6) Å and offsets of 1.52 (2) Å. Pairs of Re_2_O_2_Br_6_ anions are also linked into dimers by additional pairwise Re⋯Br contacts [Br6⋯Re2 = 3.131 (3) Å]. Cationic and anionic dimers are packed into a three-dimensional network by non-directional inter­molecular electrostatic forces and by C—H⋯Br and C—H⋯S hydrogen bonds (Table 1 ▸). Solvent-accessible channels along the *b* axis are occupied by the disordered 1,1,2-tri­chloro­ethane mol­ecules.

## Database survey   

A search of the Cambridge Structural Database (Version 5.36; last update February 2015; Groom *et al.*, 2016 ▸) for related compounds of bis­(ethyl­enedi­thio)­tetra­thia­fulvalene with simple Re-containing anions resulted in eight hits, amongst which one closely related structure containing the ET cation and Re_2_Cl_8_ anion (Reinheimer *et al.*, 2008 ▸). A search for Re_2_Hal*_x_L_y_* anionic moieties, where Hal is a halogen atom and *L* is the μ_2_-carb­oxy­lic group, resulted in nine hits. Some closely related patterns were found, *e.g.* one containing the (μ_2_-acetato)-hexa­chlorido­dirhenate anion exhibiting the same structure of the title compound (Vega *et al.*, 2002 ▸), and one containing the di-μ_2_-acetato-bis­(di­bromido­rhenate) anion (Koz’min *et al.*, 1981 ▸).

## Synthesis and crystallization   

The synthesis of the radical cation title salt was performed by galvanostatic anodic oxidation of ET (0.002 mol l^−1^) in a two-electrode U-shaped glass cell with platinum electrodes. The initial current intensity of 0.1 µA was increased by 0.05 µA per day to a final value of 0.45 µA. A mixture of 1,1,2-tri­chloro­ethane/aceto­nitrile (12:1 *v*/*v*) was used as solvent. [(C_4_H_9_)_4_N]_2_[Re_2_Br_8_] (0.008 mol l^−1^) was used as electrolyte. After a period of 6–7 weeks, black shiny plate-shaped crystals of the title salt suitable for X-ray analysis were formed.

## Refinement   

Crystal data, data collection and structure refinement details are summarized in Table 2 ▸. All hydrogen atoms were placed in idealized positions and refined using a riding-model approximation, with C—H = 0.96–0.97 Å, and with *U*_iso_(H) = 1.2*U*_eq_(C) or 1.5*U*_eq_(C) for methyl H atoms. The 1,1,2-tri­chloro­ethane mol­ecule is disordered over two sets of sites about a twofold rotation axis with equal occupancy. The C4–C5 and C9–C10 groups of the ET cations are disordered over two orientations with occupancy factors of 0.65/0.35 and 0.77/0.23, respectively. These occupancies were initially obtained as free variables by the full-matrix refinement, and were then fixed in the final refinement cycles. The C—C and C—Cl bond lengths in the solvent mol­ecule were constrained to be 1.52 (1) and 1.80 (1) Å, respectively, and the C—Cl bonds of the solvent mol­ecule were restrained to have the same lengths to within 0.01 Å. The C—S and C—C bonds of the disordered fragments of the ET cation were also restrained to have the same lengths to within 0.005 Å. The atoms of each disordered fragment, including the solvent mol­ecule, were restrained to have approximately the same displacement parameters to within 0.02–0.04 Å^2^. DELU restraints to within 0.01 Å^2^ were applied to atoms C4*B*, C5*B*, C9*B*, C10*B*, C1*S* and Cl2*S*. In addition, all non-hydrogen atoms of the solvent mol­ecule were restrained to be approximately isotropic to within 0.03–0.06 Å^2^. Several outlier reflections (67) that were believed to be affected by the contribution of several unresolved minor twin domains were omitted from the final cycles of refinement, reducing the *R* factor from 0.061 to 0.052. Attempts to refine the structure using a two-component twin model were unsuccessful. Moreover, the crystals of the title compound are stable but show a strong tendency to splicing. The poor quality of the available crystal may account for the rather low bond precision of the C—C bonds and the presence of several large residual density peaks.

## Supplementary Material

Crystal structure: contains datablock(s) I. DOI: 10.1107/S2056989016006058/rz5188sup1.cif

Structure factors: contains datablock(s) I. DOI: 10.1107/S2056989016006058/rz5188Isup2.hkl

CCDC reference: 1473493

Additional supporting information:  crystallographic information; 3D view; checkCIF report

## Figures and Tables

**Table 1:** Hydrogen-bond geometry (Å, °)

*D*—H⋯*A*	*D*—H	H⋯*A*	*D*⋯*A*	*D*—H⋯*A*
C5*B*—H5*BA*⋯Br1	0.98	2.77	3.63 (8)	147
C9*A*—H9*AA*⋯Br6^i^	0.97	2.80	3.60 (3)	140
C9*B*—H9*BA*⋯S4^ii^	0.97	2.75	3.46 (10)	130
C9*B*—H9*BB*⋯Br6^i^	0.96	2.61	3.40 (11)	140
C10*A*—H10*A*⋯Br4^iii^	0.97	2.92	3.83 (4)	156
C10*A*—H10*B*⋯S3^ii^	0.97	2.81	3.57 (3)	136
C10*B*—H10*D*⋯Br4^iii^	0.98	2.67	3.61 (11)	161

Symmetry codes: (i) 
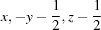
; (ii) 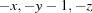; (iii) 
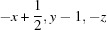
.

**Table 2:** Experimental details

Crystal data	
Chemical formula	(C_10_H_8_S_8_)[Re_2_Br_6_(C_2_H_3_O_2_)]·0.5C_2_H_3_Cl_3_
*M*_r_	1362.24
Crystal system, space group	Monoclinic, *I*2/*a*
Temperature (K)	298
*a*, *b*, *c* (Å)	27.1825 (5), 8.53737 (13), 26.0667 (5)
β (°)	100.8440 (17)
*V* (Å^3^)	5941.21 (18)
*Z*	8
Radiation type	Mo *K*α
μ (mm^−1^)	16.93
Crystal size (mm)	0.4 × 0.4 × 0.1

Data collection	
Diffractometer	Agilent Xcalibur Sapphire3
Absorption correction	Multi-scan (*CrysAlis PRO*; Agilent, 2014 ▸)
*T*_min_, *T*_max_	0.067, 1.000
No. of measured, independent and observed [*I* > 2σ(*I*)] reflections	36051, 6755, 6304
*R*_int_	0.039
(sin θ/λ)_max_ (Å^−1^)	0.650

Refinement	
*R*[*F* ^2^ > 2σ(*F* ^2^)], *wR*(*F* ^2^), *S*	0.052, 0.137, 1.14
No. of reflections	6755
No. of parameters	334
No. of restraints	99
H-atom treatment	H-atom parameters constrained
Δρ_max_, Δρ_min_ (e Å^−3^)	1.77, −1.90

Computer programs: *CrysAlis PRO* (Agilent, 2014 ▸), *SHELXS97* (Sheldrick, 2008 ▸), *SHELXL2014* (Sheldrick, 2015 ▸) and *OLEX2* (Dolomanov *et al.*, 2009 ▸).

## supplementary crystallographic information

### Crystal data

**Table:**

(C_10_H_8_S_8_)[Re_2_Br_6_(C_2_H_3_O_2_)]·0.5C_2_H_3_Cl_3_	*F*(000) = 4960
*M**_r_* = 1362.24	*D*_x_ = 3.046 Mg m^−^^3^
Monoclinic, *I*2/*a*	Mo *K*α radiation, λ = 0.71073 Å
*a* = 27.1825 (5) Å	Cell parameters from 18811 reflections
*b* = 8.53737 (13) Å	θ = 2.9–30.7°
*c* = 26.0667 (5) Å	µ = 16.93 mm^−^^1^
β = 100.8440 (17)°	*T* = 298 K
*V* = 5941.21 (18) Å^3^	Block, metallic dark violet
*Z* = 8	0.4 × 0.4 × 0.1 mm

### Data collection

**Table:**

Agilent Xcalibur Sapphire3 diffractometer	6755 independent reflections
Radiation source: Enhance (Mo) X-ray Source	6304 reflections with *I* > 2σ(*I*)
Graphite monochromator	*R*_int_ = 0.039
Detector resolution: 16.1827 pixels mm^-1^	θ_max_ = 27.5°, θ_min_ = 2.9°
ω scans	*h* = −35→35
Absorption correction: multi-scan (*CrysAlis PRO*; Agilent, 2014)	*k* = −11→9
*T*_min_ = 0.067, *T*_max_ = 1.000	*l* = −33→33
36051 measured reflections	

### Refinement

**Table:**

Refinement on *F*^2^	Primary atom site location: structure-invariant direct methods
Least-squares matrix: full	Secondary atom site location: difference Fourier map
*R*[*F*^2^ > 2σ(*F*^2^)] = 0.052	Hydrogen site location: inferred from neighbouring sites
*wR*(*F*^2^) = 0.137	H-atom parameters constrained
*S* = 1.14	*w* = 1/[σ^2^(*F*_o_^2^) + (0.0534*P*)^2^ + 229.8497*P*] where *P* = (*F*_o_^2^ + 2*F*_c_^2^)/3
6755 reflections	(Δ/σ)_max_ = 0.001
334 parameters	Δρ_max_ = 1.77 e Å^−^^3^
99 restraints	Δρ_min_ = −1.90 e Å^−^^3^

### Special details

**Table:**

Experimental. Absorption correction: *CrysAlisPro* (Agilent, 2014) Empirical absorption correction using spherical harmonics, implemented in SCALE3 ABSPACK scaling algorithm.
Geometry. All esds (except the esd in the dihedral angle between two l.s. planes) are estimated using the full covariance matrix. The cell esds are taken into account individually in the estimation of esds in distances, angles and torsion angles; correlations between esds in cell parameters are only used when they are defined by crystal symmetry. An approximate (isotropic) treatment of cell esds is used for estimating esds involving l.s. planes.
Refinement. Refinement of F^2^ against ALL reflections. The weighted R-factor wR and goodness of fit S are based on F^2^, conventional R-factors R are based on F, with F set to zero for negative F^2^. The threshold expression of F^2^ > 2sigma(F^2^) is used only for calculating R-factors(gt) etc. and is not relevant to the choice of reflections for refinement. R-factors based on F^2^ are statistically about twice as large as those based on F, and R- factors based on ALL data will be even larger.

### Fractional atomic coordinates and isotropic or equivalent isotropic displacement parameters (Å^2^)

**Table:**

	*x*	*y*	*z*	*U*_iso_*/*U*_eq_	Occ. (<1)
S1	0.0279 (2)	−0.3844 (7)	0.1248 (2)	0.0486 (13)	
S2	−0.0188 (2)	−0.1562 (6)	0.0484 (2)	0.0411 (11)	
S3	−0.0801 (3)	−0.0100 (7)	0.1157 (3)	0.0532 (14)	
S4	−0.0284 (3)	−0.2886 (8)	0.2057 (2)	0.0602 (17)	
S5	0.0889 (2)	−0.5248 (8)	0.0393 (2)	0.0484 (13)	
S6	0.0408 (2)	−0.2918 (6)	−0.0354 (2)	0.0436 (12)	
S7	0.0915 (3)	−0.3875 (7)	−0.1204 (2)	0.0586 (17)	
S8	0.1502 (3)	−0.6653 (9)	−0.0297 (2)	0.0590 (16)	
C1	0.0209 (7)	−0.313 (2)	0.0627 (7)	0.034 (4)	
C2	−0.0374 (7)	−0.152 (2)	0.1079 (8)	0.037 (4)	
C3	−0.0160 (8)	−0.262 (3)	0.1431 (8)	0.043 (5)	
C4	−0.065 (3)	−0.122 (6)	0.220 (3)	0.064 (11)	0.35
H4A	−0.1001	−0.1537	0.2165	0.077*	0.35
H4B	−0.0538	−0.0909	0.2563	0.077*	0.35
C4A	−0.0391 (15)	−0.084 (3)	0.2196 (15)	0.056 (8)	0.65
H4AA	−0.0440	−0.0750	0.2554	0.067*	0.65
H4AB	−0.0094	−0.0242	0.2166	0.067*	0.65
C5A	−0.0822 (16)	−0.015 (5)	0.1850 (9)	0.060 (9)	0.65
H5AA	−0.0864	0.0909	0.1965	0.072*	0.65
H5AB	−0.1118	−0.0737	0.1893	0.072*	0.65
C5B	−0.062 (3)	0.011 (9)	0.1862 (9)	0.061 (12)	0.35
H5BA	−0.0272	0.0463	0.1933	0.073*	0.35
H5BB	−0.0817	0.0950	0.1967	0.073*	0.35
C6	0.0472 (7)	−0.372 (2)	0.0258 (7)	0.035 (4)	
C7	0.1061 (8)	−0.523 (2)	−0.0214 (8)	0.039 (4)	
C8	0.0834 (9)	−0.415 (2)	−0.0558 (8)	0.044 (5)	
C9A	0.1534 (9)	−0.479 (4)	−0.1154 (13)	0.057 (8)	0.77
H9AA	0.1634	−0.4736	−0.1492	0.068*	0.77
H9AB	0.1777	−0.4205	−0.0908	0.068*	0.77
C9B	0.134 (4)	−0.548 (10)	−0.130 (4)	0.05 (2)	0.23
H9BA	0.1153	−0.6449	−0.1362	0.060*	0.23
H9BB	0.1493	−0.5262	−0.1596	0.060*	0.23
C10A	0.1547 (13)	−0.643 (4)	−0.0988 (9)	0.051 (7)	0.77
H10A	0.1857	−0.6902	−0.1045	0.061*	0.77
H10B	0.1272	−0.6987	−0.1202	0.061*	0.77
C10B	0.173 (4)	−0.566 (13)	−0.083 (3)	0.05 (2)	0.23
H10C	0.1860	−0.4639	−0.0715	0.059*	0.23
H10D	0.2006	−0.6266	−0.0923	0.059*	0.23
Re1	0.12639 (3)	0.02771 (9)	0.15999 (3)	0.0325 (2)	
Re2	0.18894 (3)	0.19389 (8)	0.18533 (3)	0.0285 (2)	
Br1	0.05027 (9)	0.1855 (3)	0.15459 (12)	0.0621 (7)	
Br2	0.10600 (9)	−0.1027 (3)	0.23712 (10)	0.0541 (6)	
Br3	0.11180 (11)	0.0172 (4)	0.06499 (10)	0.0623 (7)	
Br4	0.20677 (9)	0.2898 (3)	0.10198 (9)	0.0529 (6)	
Br5	0.15056 (10)	0.4496 (3)	0.19753 (11)	0.0560 (6)	
Br6	0.20369 (9)	0.1635 (3)	0.28134 (8)	0.0476 (5)	
O1	0.2381 (6)	0.0139 (18)	0.1841 (6)	0.047 (3)	
O2	0.1742 (6)	−0.1510 (17)	0.1587 (6)	0.046 (3)	
C11	0.2221 (8)	−0.129 (2)	0.1733 (7)	0.039 (4)	
C12	0.2582 (11)	−0.260 (3)	0.1768 (12)	0.066 (7)	
H12A	0.2756	−0.2551	0.1480	0.100*	
H12B	0.2404	−0.3578	0.1756	0.100*	
H12C	0.2819	−0.2533	0.2090	0.100*	
Cl1S	−0.1993 (6)	0.0569 (19)	0.0673 (6)	0.150 (6)	
Cl2S	−0.2811 (11)	0.288 (3)	0.0317 (11)	0.140 (10)	0.50
C1S	−0.2592 (9)	0.090 (4)	0.0257 (5)	0.15 (2)	
H1S	−0.2848	0.0099	0.0239	0.180*	
H1SA	−0.2718	0.1918	0.0336	0.180*	0.50

### Atomic displacement parameters (Å^2^)

**Table:**

	*U*^11^	*U*^22^	*U*^33^	*U*^12^	*U*^13^	*U*^23^
S1	0.052 (3)	0.062 (3)	0.035 (2)	0.022 (3)	0.013 (2)	0.012 (2)
S2	0.053 (3)	0.036 (2)	0.037 (2)	0.010 (2)	0.016 (2)	0.008 (2)
S3	0.064 (4)	0.044 (3)	0.055 (3)	0.018 (3)	0.022 (3)	0.001 (2)
S4	0.080 (4)	0.065 (4)	0.043 (3)	0.015 (3)	0.032 (3)	0.013 (3)
S5	0.049 (3)	0.065 (4)	0.034 (2)	0.018 (3)	0.014 (2)	0.010 (2)
S6	0.062 (3)	0.036 (2)	0.036 (2)	0.009 (2)	0.017 (2)	0.0036 (19)
S7	0.102 (5)	0.041 (3)	0.040 (3)	0.018 (3)	0.032 (3)	0.006 (2)
S8	0.064 (4)	0.075 (4)	0.042 (3)	0.028 (3)	0.020 (3)	0.008 (3)
C1	0.033 (9)	0.043 (10)	0.028 (8)	0.000 (8)	0.008 (7)	0.002 (7)
C2	0.040 (10)	0.038 (10)	0.036 (9)	−0.002 (8)	0.015 (8)	0.000 (8)
C3	0.049 (11)	0.050 (12)	0.033 (9)	0.010 (9)	0.015 (8)	0.007 (9)
C4	0.071 (17)	0.065 (16)	0.061 (15)	0.002 (14)	0.025 (14)	−0.003 (12)
C4A	0.070 (18)	0.053 (15)	0.057 (15)	0.000 (14)	0.040 (14)	−0.015 (13)
C5A	0.074 (19)	0.059 (17)	0.058 (15)	0.008 (16)	0.043 (14)	−0.007 (14)
C5B	0.066 (18)	0.061 (16)	0.060 (16)	0.000 (14)	0.023 (13)	−0.004 (12)
C6	0.039 (10)	0.039 (10)	0.030 (8)	−0.002 (8)	0.011 (7)	0.002 (7)
C7	0.042 (10)	0.045 (11)	0.031 (9)	0.004 (8)	0.012 (8)	−0.004 (8)
C8	0.067 (14)	0.036 (10)	0.030 (9)	−0.010 (10)	0.014 (9)	−0.007 (8)
C9A	0.07 (2)	0.061 (19)	0.050 (17)	−0.023 (17)	0.030 (16)	−0.004 (15)
C9B	0.05 (2)	0.05 (3)	0.05 (2)	0.000 (17)	0.013 (15)	0.001 (17)
C10A	0.058 (18)	0.050 (17)	0.050 (16)	0.014 (15)	0.025 (14)	0.003 (14)
C10B	0.05 (2)	0.05 (3)	0.05 (2)	0.000 (17)	0.013 (15)	−0.001 (17)
Re1	0.0300 (4)	0.0326 (4)	0.0333 (4)	−0.0033 (3)	0.0025 (3)	0.0011 (3)
Re2	0.0294 (4)	0.0276 (3)	0.0275 (3)	−0.0007 (2)	0.0027 (3)	0.0040 (2)
Br1	0.0443 (12)	0.0641 (15)	0.0752 (17)	0.0068 (11)	0.0042 (11)	0.0085 (13)
Br2	0.0564 (13)	0.0555 (13)	0.0517 (12)	−0.0093 (10)	0.0140 (10)	0.0070 (10)
Br3	0.0602 (15)	0.0809 (18)	0.0408 (11)	−0.0144 (13)	−0.0036 (10)	−0.0037 (11)
Br4	0.0481 (12)	0.0703 (15)	0.0389 (11)	−0.0074 (11)	0.0047 (9)	0.0135 (10)
Br5	0.0561 (13)	0.0389 (11)	0.0737 (16)	0.0048 (10)	0.0142 (12)	0.0065 (11)
Br6	0.0513 (12)	0.0517 (12)	0.0388 (10)	−0.0108 (10)	0.0056 (9)	0.0051 (9)
O1	0.050 (9)	0.041 (8)	0.048 (8)	0.002 (7)	0.005 (7)	0.004 (7)
O2	0.056 (9)	0.034 (7)	0.048 (8)	−0.001 (6)	0.009 (7)	−0.004 (6)
C11	0.056 (12)	0.033 (9)	0.031 (9)	0.005 (9)	0.014 (8)	−0.001 (8)
C12	0.074 (18)	0.048 (14)	0.085 (19)	0.023 (13)	0.034 (15)	0.014 (13)
Cl1S	0.133 (11)	0.148 (12)	0.153 (12)	0.021 (9)	−0.014 (9)	−0.014 (10)
Cl2S	0.15 (2)	0.115 (17)	0.14 (2)	0.048 (16)	−0.024 (17)	0.005 (15)
C1S	0.15 (4)	0.12 (2)	0.19 (4)	0.02 (3)	0.05 (3)	−0.01 (3)

### Geometric parameters (Å, º)

**Table:**

S1—C1	1.708 (19)	C7—C8	1.35 (3)
S1—C3	1.72 (2)	C9A—H9AA	0.9700
S2—C1	1.71 (2)	C9A—H9AB	0.9700
S2—C2	1.72 (2)	C9A—C10A	1.46 (5)
S3—C2	1.72 (2)	C9B—H9BA	0.9700
S3—C5A	1.82 (2)	C9B—H9BB	0.9700
S3—C5B	1.82 (2)	C9B—C10B	1.46 (14)
S4—C3	1.74 (2)	C10A—H10A	0.9700
S4—C4	1.82 (2)	C10A—H10B	0.9700
S4—C4A	1.82 (2)	C10B—H10C	0.9700
S5—C6	1.72 (2)	C10B—H10D	0.9700
S5—C7	1.73 (2)	Re1—Re2	2.2174 (10)
S6—C6	1.716 (19)	Re1—Br1	2.451 (3)
S6—C8	1.72 (2)	Re1—Br2	2.451 (2)
S7—C8	1.75 (2)	Re1—Br3	2.435 (3)
S7—C9A	1.84 (2)	Re1—O2	2.009 (15)
S7—C9B	1.84 (2)	Re2—Br4	2.454 (2)
S8—C7	1.75 (2)	Re2—Br5	2.465 (2)
S8—C10A	1.84 (2)	Re2—Br6	2.473 (2)
S8—C10B	1.84 (2)	Re2—O1	2.040 (16)
C1—C6	1.40 (3)	O1—C11	1.30 (3)
C2—C3	1.36 (3)	O2—C11	1.30 (3)
C4—H4A	0.9700	C11—C12	1.48 (3)
C4—H4B	0.9700	C12—H12A	0.9600
C4—C5B	1.46 (4)	C12—H12B	0.9600
C4A—H4AA	0.9700	C12—H12C	0.9600
C4A—H4AB	0.9700	Cl1S—C1S	1.800 (16)
C4A—C5A	1.46 (4)	Cl2S—C1S	1.81 (2)
C5A—H5AA	0.9700	Cl2S—H1SA	0.8557
C5A—H5AB	0.9700	C1S—C1S^i^	1.515 (18)
C5B—H5BA	0.9700	C1S—H1S	0.9700
C5B—H5BB	0.9700	C1S—H1SA	0.9703
			
C1—S1—C3	94.9 (10)	C10A—C9A—H9AA	108.9
C1—S2—C2	95.6 (9)	C10A—C9A—H9AB	108.9
C2—S3—C5A	104.3 (14)	S7—C9B—H9BA	109.6
C2—S3—C5B	97 (3)	S7—C9B—H9BB	109.6
C5A—S3—C5B	19 (3)	H9BA—C9B—H9BB	108.1
C3—S4—C4	108 (2)	C10B—C9B—S7	110 (7)
C3—S4—C4A	97.4 (15)	C10B—C9B—H9BA	109.6
C4A—S4—C4	25 (3)	C10B—C9B—H9BB	109.6
C6—S5—C7	94.9 (9)	S8—C10A—H10A	109.0
C6—S6—C8	94.9 (10)	S8—C10A—H10B	109.0
C8—S7—C9A	98.9 (13)	C9A—C10A—S8	113 (2)
C8—S7—C9B	103 (3)	C9A—C10A—H10A	109.0
C9A—S7—C9B	26 (4)	C9A—C10A—H10B	109.0
C7—S8—C10A	102.8 (12)	H10A—C10A—H10B	107.8
C7—S8—C10B	96 (4)	S8—C10B—H10C	109.1
C10B—S8—C10A	28 (4)	S8—C10B—H10D	109.1
S1—C1—S2	116.1 (11)	C9B—C10B—S8	112 (7)
C6—C1—S1	122.7 (15)	C9B—C10B—H10C	109.1
C6—C1—S2	121.1 (15)	C9B—C10B—H10D	109.1
S3—C2—S2	116.0 (12)	H10C—C10B—H10D	107.9
C3—C2—S2	115.7 (15)	Re2—Re1—Br1	104.91 (8)
C3—C2—S3	128.3 (16)	Re2—Re1—Br2	108.99 (7)
S1—C3—S4	116.6 (12)	Re2—Re1—Br3	107.19 (7)
C2—C3—S1	117.5 (15)	Br1—Re1—Br2	88.73 (10)
C2—C3—S4	125.9 (17)	Br3—Re1—Br1	89.29 (11)
S4—C4—H4A	109.2	Br3—Re1—Br2	143.01 (9)
S4—C4—H4B	109.2	O2—Re1—Re2	91.6 (4)
H4A—C4—H4B	107.9	O2—Re1—Br1	163.4 (4)
C5B—C4—S4	112 (5)	O2—Re1—Br2	85.2 (5)
C5B—C4—H4A	109.2	O2—Re1—Br3	86.4 (4)
C5B—C4—H4B	109.2	Re1—Re2—Br4	102.59 (6)
S4—C4A—H4AA	108.8	Re1—Re2—Br5	106.59 (7)
S4—C4A—H4AB	108.8	Re1—Re2—Br6	101.74 (6)
H4AA—C4A—H4AB	107.7	Br4—Re2—Br5	88.72 (9)
C5A—C4A—S4	114 (3)	Br4—Re2—Br6	155.47 (8)
C5A—C4A—H4AA	108.8	Br5—Re2—Br6	87.39 (9)
C5A—C4A—H4AB	108.8	O1—Re2—Re1	88.9 (4)
S3—C5A—H5AA	108.1	O1—Re2—Br4	89.9 (5)
S3—C5A—H5AB	108.1	O1—Re2—Br5	164.3 (4)
C4A—C5A—S3	117 (2)	O1—Re2—Br6	87.4 (5)
C4A—C5A—H5AA	108.1	C11—O1—Re2	120.9 (14)
C4A—C5A—H5AB	108.1	C11—O2—Re1	119.9 (13)
H5AA—C5A—H5AB	107.3	O1—C11—C12	120 (2)
S3—C5B—H5BA	107.3	O2—C11—O1	118.4 (18)
S3—C5B—H5BB	107.3	O2—C11—C12	121 (2)
C4—C5B—S3	120 (5)	C11—C12—H12A	109.5
C4—C5B—H5BA	107.3	C11—C12—H12B	109.5
C4—C5B—H5BB	107.3	C11—C12—H12C	109.5
H5BA—C5B—H5BB	106.9	H12A—C12—H12B	109.5
S6—C6—S5	116.3 (11)	H12A—C12—H12C	109.5
C1—C6—S5	122.5 (15)	H12B—C12—H12C	109.5
C1—C6—S6	121.2 (15)	C1S—Cl2S—H1SA	8.9
S5—C7—S8	114.5 (12)	Cl1S—C1S—Cl2S	112 (2)
C8—C7—S5	116.4 (16)	Cl1S—C1S—H1S	118.6
C8—C7—S8	129.0 (16)	Cl1S—C1S—H1SA	109.2
S6—C8—S7	115.3 (13)	Cl2S—C1S—H1S	114.7
C7—C8—S6	117.5 (16)	Cl2S—C1S—H1SA	7.8
C7—C8—S7	127.2 (18)	C1S^i^—C1S—Cl1S	97 (2)
S7—C9A—H9AA	108.9	C1S^i^—C1S—Cl2S	104.2 (12)
S7—C9A—H9AB	108.9	C1S^i^—C1S—H1S	108.0
H9AA—C9A—H9AB	107.7	C1S^i^—C1S—H1SA	112.0
C10A—C9A—S7	114 (2)	H1S—C1S—H1SA	111.3
			
S1—C1—C6—S5	0 (3)	C7—S8—C10A—C9A	40 (3)
S1—C1—C6—S6	−177.4 (11)	C7—S8—C10B—C9B	−63 (7)
S2—C1—C6—S5	177.7 (11)	C8—S6—C6—S5	0.2 (14)
S2—C1—C6—S6	0 (2)	C8—S6—C6—C1	178.1 (17)
S2—C2—C3—S1	2 (3)	C8—S7—C9A—C10A	58 (3)
S2—C2—C3—S4	−178.8 (14)	C8—S7—C9B—C10B	−42 (7)
S3—C2—C3—S1	−177.1 (13)	C9A—S7—C8—S6	158.8 (15)
S3—C2—C3—S4	2 (3)	C9A—S7—C8—C7	−22 (2)
S4—C4—C5B—S3	58 (9)	C9A—S7—C9B—C10B	42 (6)
S4—C4A—C5A—S3	−61 (4)	C9B—S7—C8—S6	−175 (4)
S5—C7—C8—S6	1 (2)	C9B—S7—C8—C7	4 (4)
S5—C7—C8—S7	−178.2 (13)	C9B—S7—C9A—C10A	−43 (8)
S7—C9A—C10A—S8	−71 (3)	C10A—S8—C7—S5	174.5 (16)
S7—C9B—C10B—S8	77 (9)	C10A—S8—C7—C8	−4 (3)
S8—C7—C8—S6	179.3 (13)	C10A—S8—C10B—C9B	43 (6)
S8—C7—C8—S7	0 (3)	C10B—S8—C7—S5	−158 (4)
C1—S1—C3—S4	176.8 (14)	C10B—S8—C7—C8	23 (4)
C1—S1—C3—C2	−4 (2)	C10B—S8—C10A—C9A	−40 (8)
C1—S2—C2—S3	−179.7 (12)	Re1—Re2—O1—C11	−3.2 (15)
C1—S2—C2—C3	0.9 (19)	Re1—O2—C11—O1	−6 (2)
C2—S2—C1—S1	−3.7 (13)	Re1—O2—C11—C12	175.2 (17)
C2—S2—C1—C6	178.7 (17)	Re2—Re1—O2—C11	3.0 (15)
C2—S3—C5A—C4A	22 (4)	Re2—O1—C11—O2	6 (3)
C2—S3—C5B—C4	−61 (7)	Re2—O1—C11—C12	−175.0 (17)
C3—S1—C1—S2	4.6 (14)	Br1—Re1—Re2—Br4	−90.90 (10)
C3—S1—C1—C6	−177.8 (18)	Br1—Re1—Re2—Br5	1.54 (11)
C3—S4—C4—C5B	−19 (7)	Br1—Re1—Re2—Br6	92.30 (10)
C3—S4—C4A—C5A	63 (3)	Br1—Re1—Re2—O1	179.4 (5)
C4—S4—C3—S1	168 (3)	Br1—Re1—O2—C11	−174.7 (11)
C4—S4—C3—C2	−11 (4)	Br2—Re1—Re2—Br4	175.23 (10)
C4—S4—C4A—C5A	−54 (6)	Br2—Re1—Re2—Br5	−92.32 (10)
C4A—S4—C3—S1	144.9 (18)	Br2—Re1—Re2—Br6	−1.56 (10)
C4A—S4—C3—C2	−34 (3)	Br2—Re1—Re2—O1	85.6 (5)
C4A—S4—C4—C5B	50 (4)	Br2—Re1—O2—C11	−105.9 (15)
C5A—S3—C2—S2	−169.7 (19)	Br3—Re1—Re2—Br4	3.05 (11)
C5A—S3—C2—C3	10 (3)	Br3—Re1—Re2—Br5	95.49 (11)
C5A—S3—C5B—C4	53 (6)	Br3—Re1—Re2—Br6	−173.75 (10)
C5B—S3—C2—S2	−152 (3)	Br3—Re1—Re2—O1	−86.6 (5)
C5B—S3—C2—C3	28 (3)	Br3—Re1—O2—C11	110.1 (15)
C5B—S3—C5A—C4A	−48 (8)	Br4—Re2—O1—C11	−105.8 (15)
C6—S5—C7—S8	−179.3 (12)	Br5—Re2—O1—C11	169.2 (11)
C6—S5—C7—C8	−0.5 (19)	Br6—Re2—O1—C11	98.6 (15)
C6—S6—C8—S7	178.5 (13)	O2—Re1—Re2—Br4	89.8 (5)
C6—S6—C8—C7	−0.6 (19)	O2—Re1—Re2—Br5	−177.8 (5)
C7—S5—C6—S6	0.1 (14)	O2—Re1—Re2—Br6	−87.0 (5)
C7—S5—C6—C1	−177.7 (18)	O2—Re1—Re2—O1	0.1 (6)

Symmetry code: (i) −*x*−1/2, *y*, −*z*.

### Hydrogen-bond geometry (Å, º)

**Table:**

*D*—H···*A*	*D*—H	H···*A*	*D*···*A*	*D*—H···*A*
C5*B*—H5*BA*···Br1	0.98	2.77	3.63 (8)	147
C9*A*—H9*AA*···Br6^ii^	0.97	2.80	3.60 (3)	140
C9*B*—H9*BA*···S4^iii^	0.97	2.75	3.46 (10)	130
C9*B*—H9*BB*···Br6^ii^	0.96	2.61	3.40 (11)	140
C10*A*—H10*A*···Br4^iv^	0.97	2.92	3.83 (4)	156
C10*A*—H10*B*···S3^iii^	0.97	2.81	3.57 (3)	136
C10*B*—H10*D*···Br4^iv^	0.98	2.67	3.61 (11)	161

Symmetry codes: (ii) *x*, −*y*−1/2, *z*−1/2; (iii) −*x*, −*y*−1, −*z*; (iv) −*x*+1/2, *y*−1, −*z*.
